# Experimental Investigation on the Mechanical and Physical Properties of Glass/Jute Hybrid Laminates

**DOI:** 10.3390/polym14214742

**Published:** 2022-11-05

**Authors:** Thaís da Costa Dias, Amanda Albertin Xavier da Silva, Maikson Luiz Passaia Tonatto, Sandro Campos Amico

**Affiliations:** 1 Post-Graduate Program in Mining, Metallurgical and Materials Engineering, Federal University of Rio Grande do Sul, Porto Alegre 91501-970, Brazil; 2 Post-Graduate Program in Mechanical Engineering, Federal University of Santa Maria, Cachoeira do Sul 96503-205, Brazil

**Keywords:** natural fibres, hybrid composite, mechanical and physical properties, laminates

## Abstract

Natural fibres have been partly substituting synthetic fibres in polymer composites due to their renewable character and many other advantages, and sometimes, they can be hybridized into a single composite for a better combination of properties. This work aims to study the effect of hybridization and stacking sequence on the mechanical and physical properties of the glass/jute laminates. For that, pure jute, pure glass and glass/jute hybrids were manufactured by vacuum infusion process using orthophthalic polyester resin. The composites were characterized via C-scan analysis, density, volume fraction of constituents and optical microscopy analyses. Mechanical properties were obtained from tensile, compression and shear tests. The longitudinal properties were higher than transverse properties for all laminates. The hybrids presented intermediate density and mechanical properties compared to pure glass and pure jute laminates. The hybrids produced similar density and tensile modulus, but with small differences in tensile strength and compressive strength which were justified based on variations in resin and void content due to the influence of the stacking sequence (glass/jute interlayer regions). In addition, the pure glass and the hybrid laminates displayed acceptable failure morphology in the in-plane shear test, but not the pure jute laminate.

## 1. Introduction

With the increase in environmental consciousness, community interest, and regulations, the use of environmentally friendly materials is constantly increasing [1,2]. Natural fibre-reinforced polymer composites are attractive in diversified applications from household to aeronautical due to their light weight, biodegradability, renewability, high specific strength and stiffness, good corrosion resistivity, enhanced energy recovery, and lower fabrication cost, among others [3,4,5,6,7].

Therefore, plant fibres are an interesting alternative for use in composites, partly substituting synthetic fibres [8]. In particular, the jute fibre is one of the longest natural fibres, being largely grown in Bangladesh, India and China. Jute is also one of the cheap lignocellulosic fibres, easily available in fibre and fabric form, with mean constitution of cellulose (61–72%), hemicellulose (14–20.4%), lignin (12–13%), and pectin (0.2%) [6,9,10,11].

Jute fibre has been studied as a replacement for synthetic fibres, such as glass fibres, in many composite applications, focusing on their interfacial, physical, and mechanical properties [12]. The compressive and tensile behaviour of unidirectional natural fibres composites was studied by Baley et al. [13] through a parametric analysis considering the nature of the fibre (flax or jute), the matrix (thermoplastic and thermoset: PP, PP/MAPP, PA11, epoxy or acrylic), the fibre content, and the fibre/matrix interfacial strength. In [14], compressed unidirectional raw jute fibre sheets produced from raw jute reed and jute silver were used for the preparation of composites with polyester and having different fibre content. The tensile, flexural and interlaminar shear strengths of the composites made from raw jute were higher than those from jute silver [14].

An experimental and theoretical study was carried out to determine longitudinal Young’s modulus of unidirectional laminate jute/epoxy composite [15]. The experimental and analytical results were in a good agreement, and a higher fibre content was found critical for obtaining the desired mechanical properties. In another work, a novel jute-yarn, non-crimp, unidirectional (UD) preform, with three different types of warp jute yarns of varying linear densities and twists was investigated [16]. Polyester resin was used as matrix and the composites were manufactured by compression moulding. The obtained tensile strength varied within 94–133 MPa and the tensile modulus within 5.6–8.3 GPa. Another aspect evaluated in composites with woven jute was the effect of the gamma radiation on jute, which was found to improve some mechanical properties such as tensile strength and modulus [17].

One of the main disadvantages of natural fibres as a reinforcing material is its hydrophilic nature, responsible for moisture absorption, which may cause swelling and maceration of fibres, thus significantly decreasing their mechanical properties [10]. Jute fibre absorbs moisture to a great extent, and has poor wettability by an organic matrix resin, resulting in weak interfacial bonding between fibres and resins such as polyester, epoxy, and phenolic [18,19].

Hybridization of synthetic fibres with natural ones minimizes the overall production cost and makes the hybrid system more tolerant to damage, especially to brittle failure, and is more environmentally friendly [20]. Glass fibre has much lower cost than carbon fibre and, to keep this advantage, it can be combined with other low-cost fibres such as natural ones in a hybrid composite, with a new set of properties suitable for various applications. Glass fibre is also a strong and tough material, improving overall mechanical properties [21,22].

Researchers have studied the hybridization of jute with synthetic fibres, which would reduce known disadvantages of natural fibres such as low moisture resistance, poor wettability and poor dimensional stability. In [23], jute/E-glass (30% fibre content) composites manufactured by hand lay-up yielded greater mechanical and wear properties than single-fibre composites [23], whereas [8] produced composites by hand lay-up with a combination of jute–glass and two resins (epoxy–polyester) and found the highest tensile strength for the jute–glass fibre reinforced 50%E–50%P hybrid composite among the studied combinations [24]. In another work, glass–jute hybrid composites with different stacking sequences were prepared by hand lay-up. The tensile properties of the glass fibre composite were only slightly affected at low jute fibre content, i.e., the strength of composites consisting of one jute and four glass laminas were comparable with that of five glass laminas, with an associated cost advantage. Numerical simulation was also carried out. Deviations between experimental and numerical results were found for different stacking sequences, which were attributed to non-uniformity in jute fibre diameter and to the manufacturing process used. In addition, fractographic analysis revealed micro-voids and adhesive failure at different layers of fibres as the primary cause of delamination [25].

The effect of ply sequence on the mechanical properties of composites with synthetic fibres has been widely investigated, for example, in [26,27]. As for natural fibres, the effect of stacking sequency and ply orientation was investigated in some works, e.g., hybrid composite of pineapple leaf fibre (PALF) and carbon fibre [28].

Other works include those of Ouarhim et al. [29], who investigated moisture absorption and mechanical properties of bidirectional fabrics of jute and glass inter-layer (layer-by-layer) and intra-layer (yarn-by-yarn) hybridization configurations, and of Sujon et al. [30], who studied the influence of fibre orientation and stacking sequence on mechanical and water absorption properties of jute/carbon. In addition, the hybridization of jute fibres with other natural fibres was studied in [31,32].

The literature review showed limited work on unidirectional jute laminates and their hybridization with glass fibres, and most of them addressed only tensile properties and limited to the longitudinal direction. In this context, the present work focuses on pure glass, pure jute and glass–jute hybrid laminates, investigating the effect of hybridization and stacking sequence on general mechanical and physical properties of those composites.

## 2. Materials and Methods

### 2.1. Materials and Manufacturing

Orthophthalic polyester resin (Alpha 163) supplied by Induspol Industria de Polimeros (Jandira/SãoPaulo/Brazil) with 1% of initiator Butanox M50 was used. The reinforcements were a Vew 090/50 (E-glass, mostly unidirectional) fabric (450 g/m^2^ aerial weight) from Owens Corning and a unidirectional jute fabric (225 g/m^2^ aerial weight) supplied by Textil P.B.S. (Nova Odessa/São Paulo/Brazil). The jute fabrics underwent oven drying for 60 min at 105 °C for moisture removal prior to moulding.

Compressibility of the dry fabrics was evaluated in a Universal Instron testing machine (model 3382) using circular parallel compression plates, as in [33]. The test was started by placing the stack of fabrics between the plates spaced by 5 mm. The compression load (in turn, pressure in kPa) required to bring the reinforcement to a particular thickness, representing a specific fibre volume fraction, was recorded.

To obtain the composite, the fabrics were cut and stacked in a lay-up (600 × 300) mm on a one-sided mould, followed by a layer of peel ply and a flow mesh which partly covered the fabrics. The mould was sealed using tacky tape and vacuum bag. The cavity was then evacuated (0.6 bar), removing air and compacting the reinforcement. The resin infiltrated the perform and was allowed to cure for 24 h under vacuum. Later, the composite was extracted and post-cured (3 h at 60 °C). The laminates were later machined using a CNC cutter to obtain specimens for testing.

**Figure 1:**
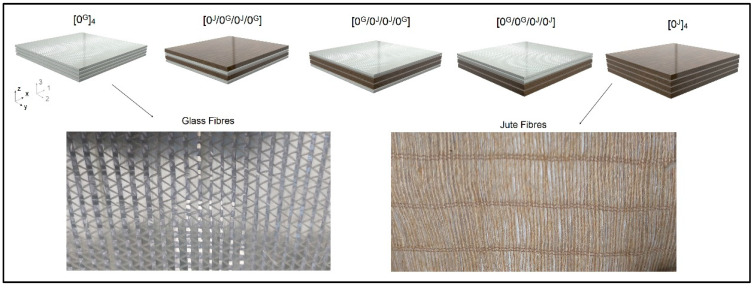
Stacking sequence and nomenclature adopted for the studied laminates.

Five laminates were manufactured. Figure 1 depicts the stacking sequences and the nomenclature adopted for them. All laminates are comprised of four layers and there are three hybrid conditions.

### 2.2. Laminate Characterization and Properties

C-scan ultrasonic inspection was performed using an NDT Systems equipment, Raptor model, with 2.25 MHz (0.5 inch) transducer and 35 dB gain with water as coupling medium to observe resin distribution and homogeneity. Higher values of amplitude scale (toward yellow and green tones) indicate higher resin content, and lower values (toward darker tones) indicate higher content of fibres and voids.

A Carl Zeiss Lab A model optical microscope was used to analyse the cross section of the laminates and to obtain overall and layer thicknesses. A Zoom Microscope 1600× Cam 2.0 Mp Professional was used to further analyse fracture of the pure jute sample after shear testing.

Density of the laminates was evaluated according to ASTM D792 standard, which is based on the Archimedes’ principle, and three specimens were used for each condition. The overall fibre volume fraction (*V_f_*), and the jute (*V_J_*), glass (*V_G_*), matrix (*V_m_*), and void (*V_v_*) volume fractions, along with matrix mass fraction (*W_m_*), were determined analytically based on [34]:

(1) $$V_{f}=\frac{1}{t}\left(\frac{N_{G}S_{G}}{\rho_{G}}+\frac{N_{J}S_{J}}{\rho_{J}}\right),\;V_{m}=W_{m}\frac{\rho_{c}}{\rho_{m}},\;V_{v}=100-\left(V_{f}+V_{m}\right),$$

where *N* is the number of layers, *t* is the total thickness, *S* is fabric aerial density, *ρ* is density, and the subscripts *G*, *J* and *C* refer to jute, glass, and composite, respectively. In the case of hybrids, for the calculation of individual fibre fractions, the distributive property was used in the equation of the fibre volume fraction. The density considered for jute fibre was 1.46 g/cm^3^ and for glass fibre was 2.5 g/cm^3^ [35]. For the laminates with only glass fibres, the fibre weight content was directly determined by matrix thermal degradation (temperature ≈ 600 °C, digestion time ≈ 6 h) according to ASTM D3171.

The pure polyester resin was tensile tested according to ASTM D638 in a Instron machine with 5 mm/min and a 100 kN load-cell. The strain was measured with a video extensometer. Tensile test of the laminates was performed according to ASTM D3039, with 2 mm/min displacement ratio, with clip-gauge extensometers in the x and y directions to obtain longitudinal and transverse strains, respectively. For each condition, five specimens were used, and the properties were obtained in the longitudinal (0°) and transverse (90°) fibre directions. The modulus was calculated from 0.1 to 0.3% strain. The major Poisson’s ratio (*ν*_12_) was experimentally determined, and the minor Poisson’s ratio (*ν*_21_) was calculated using the $\frac{\nu_{12}}{E_{1}}=\frac{\nu_{21}}{E_{2}}$ correlation [34].

Compressive testing was carried out according to ASTM D6641 in a Instron, at 1.3 mm/min displacement ratio, also in the longitudinal and transverse directions, to obtain the compressive strengths. Finally, shear testing was performed according to ASTM 7078 at 2 mm/min displacement ratio (Instron) to obtain shear stress at 5% strain.

The experimental data were analysed via Analysis of Variance (ANOVA) and Tukey’s test using Minitab Software v.17 within a 95% confidence interval. The letters represent the results obtained by the Tukey’s comparison test, and similar letters in a particular group indicate that there is no significant difference among the means.

The engineering constants of the studied symmetric hybrid laminate ([0^G^/0^J^/0^J^/0^G^]) were estimated using the correlations shown in [34], where *E_x_* and *E_y_* are the longitudinal and transverse modulus, respectively, *G_xy_* is the shear modulus, *ν_xy_* and *ν_yx_* are the major and minor Poisson’s ratio, respectively, *t* is thickness of the laminate, and A* are the terms of the ABD^−1^ matrix, calculated using the MECH-Gcomp software v. 1.11 [36] based on the experimental elastic properties for the pure jute and pure glass laminates.

## 3. Results and Discussion

### 3.1. Physical Properties

**Figure 2:**
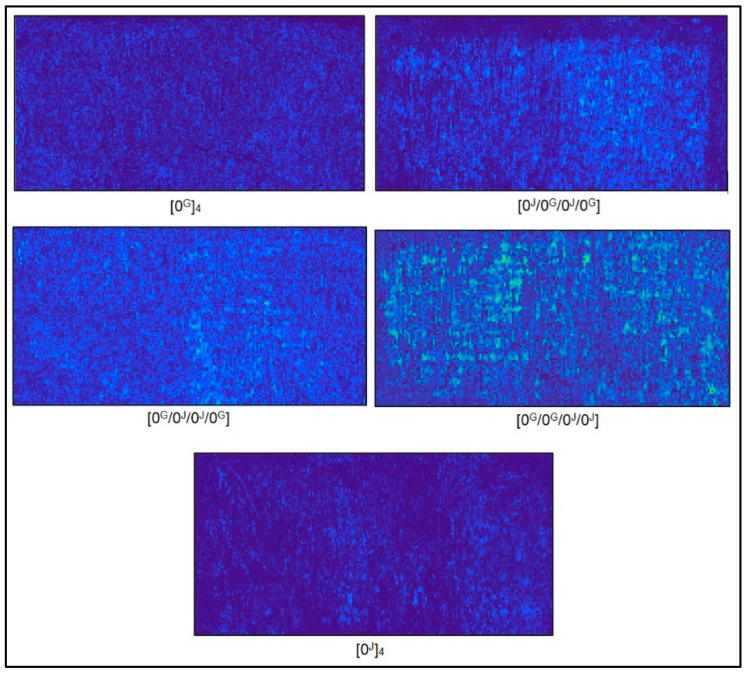
C-scan maps obtained for the studied laminates.

Figure 2 shows the results from the C-scan analyses for all laminates, which showed reasonably uniform resin distribution. The [0^J^/0^G^/0^J^/0^G^] and [0^G^/0^G^/0^J^/0^J^] showed a higher colour contrast and higher concentration of bright spots, indicating slightly richer resin spots, i.e., lower overall quality. On the other hand, [0^G^/0^J^/0^J^/0^G^] showed greater homogeneity among the hybrids. The performance of the composite was somewhat compromised by the high content of voids, mainly strength, so a lower void content is desirable.

Table 1 shows the calculated (Equation (1)) overall fibre content (*V_f_*), and the jute (*V_J_*), glass (*V_G_*), matrix (*V_m_*), and void (*V_v_*) volume. The pure glass laminate reached the highest volume of fibres (54.14%), and the pure jute laminate the highest matrix (56.41%) and void (15.59%) volume fraction. The *V_v_* for the [0^G^]_4_ laminate, obtained directly from the thermal degradation, was 8.9% (±0.2), which is close to the value in Table 1 (8.55%) obtained from density measurements. The void and fibres content were similar among the hybrids, being slightly worse for the [0^G^/0^G^/0^J^/0^J^], corroborating the previous C-scan analysis.

Composites with natural fibres are more prone to a greater void content due to type of fibres itself, with high level of moisture and poor wettability. The volume fraction of fibre also significantly affects the composite performance, and a higher volume of fibres typically results in improved mechanical properties. Therefore, by hybridizing the jute with glass fibres, it was possible to obtain composites with higher overall fibre content and lower void content in relation to the pure jute composite.

Fiore and co-workers [37] investigated the void content for hybrid composite manufactured by vacuum infusion process with woven flax and woven glass fabrics and epoxy resin. The flax fabrics were in the middle of the laminate and the glass fabrics as the outer layers, and the void content found was 6.1%. According to Mehdikhani and co-workers [38], in the liquid composite moulding there are several causes for void formation such as mechanical air entrapment during resin flow (main cause), gas created due to chemical reactions during cure, and nucleation of dissolved gases in the resin. The air entrapment is mainly due to the inhomogeneous fibre architecture, resulting in non-uniform permeability of the fibre preform, which causes local variation in resin velocity. This local velocity variation is exacerbated by the capillary effect, prevailing at the micro-scale. The values found in the present work are higher than those of Fiore and co-workers [37], but it must be taken into account that the resin and the fabric architecture were very different.

**Figure 3:**
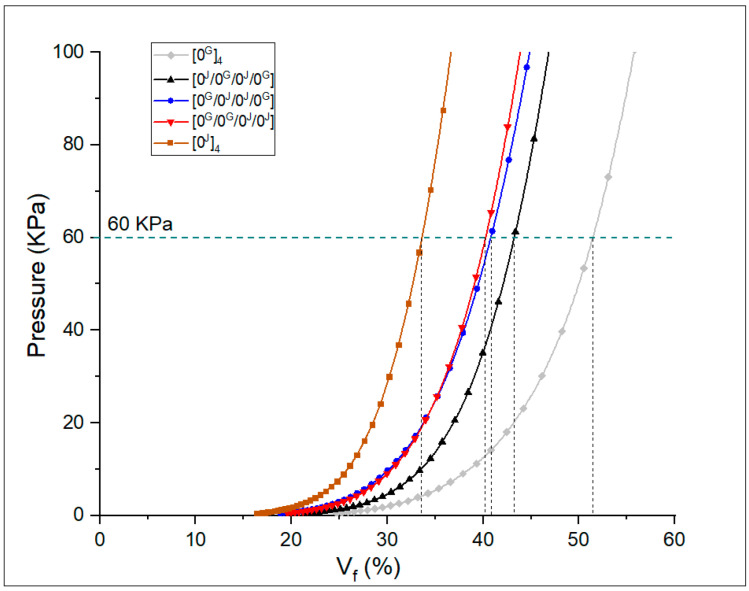
Pressure vs. predicted fibre volume fraction of the fabric preforms.

Figure 3 shows the correlation between fibre volume fraction and pressure in the fabric compressive test for the five stacking sequences. The negative pressure used in the vacuum infusion process was 0.6 bar (60 KPa), so the presented curves may be used to assess the expected fibre content in each laminate. The hybrids showed intermediate values between pure jute and pure glass, as expected by Table 1. For all laminates, except pure glass, the predicted volume fraction was slightly higher (with differences ranging from 2.5% to 5.8%) than the one shown in Table 1. Besides that, [0^G^/0^J^/0^J^/0^G^] and [0^G^/0^G^/0^J^/0^J^] were very similar, whereas [0^J^/0^G^/0^J^/0^G^] was higher. Overall, however, the difference was small.

**Figure 4:**
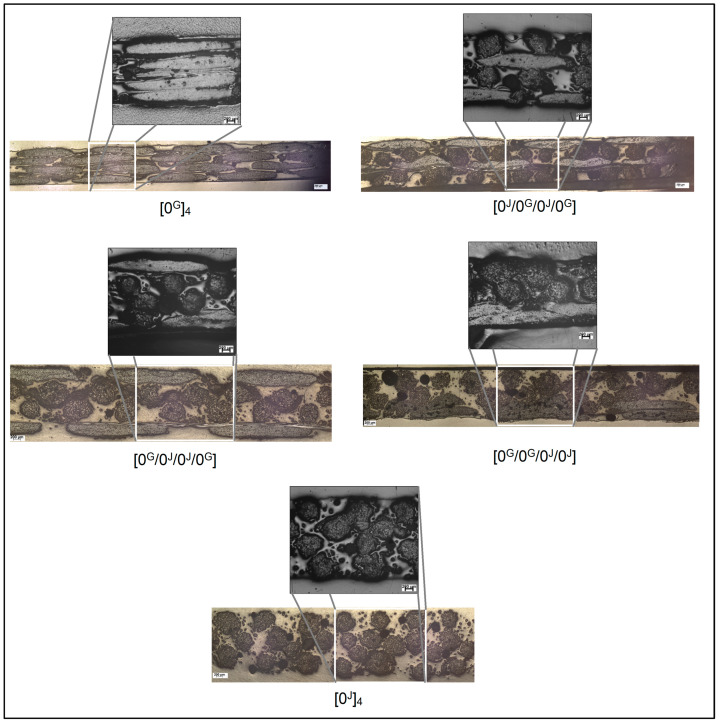
Panoramic and zoom-in images from optical microscopy for the laminates.

Figure 4 shows images from optical microscopy for the five laminates. The panoramic images show a larger portion of the cross section of the laminates and in the zoomed-in images the voids are more evident. It is possible to observe that the jute bundle is bulkier than the glass bundle and clearly the glass layers allow better packing than the jute fabric used, reducing the thickness of the laminate and the amount of matrix, also contributing to void reduction. The hybrid conditions are intermediate between pure glass and pure jute.

The micrographs in Figure 4 were also used to measure thickness (*t*) of each lamina in the laminates, and these values are compiled in Table 2 along with the overall thickness of the laminates. Pure jute laminate is the thickest, followed by the hybrids and the pure glass laminate, as expected considering the different fabric compressibility. The overall mean thickness of the jute layers is 0.58 mm, and 0.31 mm for the glass layers.

**Figure 5:**
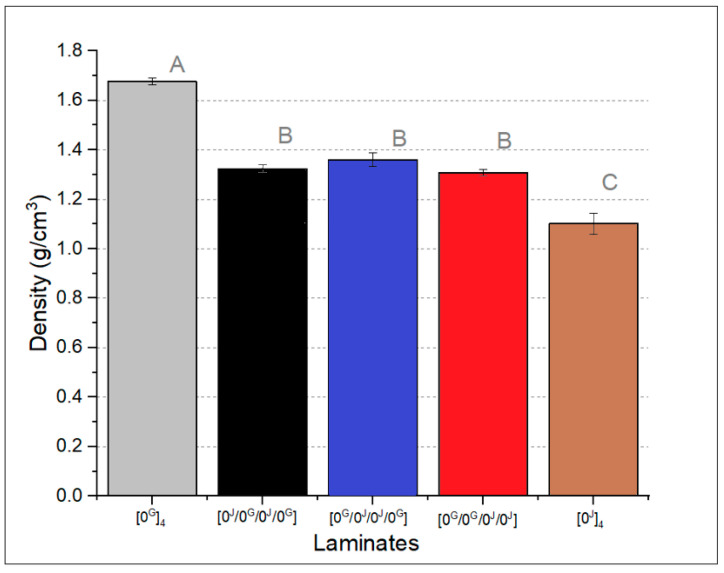
Measured density of the laminates (letters represent the results of the Tukey’s test).

For the coming results, the *p*-values from the ANOVA analyses were less than 0.05, which reveals that the stacking sequence significantly affects the evaluated properties. The letters in the columns indicate the results from the Tukey’s Test. In Figure 5, the measured density of the laminates is shown. The glass laminate presents higher density, followed by the hybrids and the jute laminate, which is expected considering the higher density of the glass fibres. There is no difference among the hybrids (same letter in the Tukey’s result) which is also expected since they have the same number of jute and glass layers. The pure jute laminate has lower density than the pure polyester (1.19 g/cm^3^ measured by Arquimedes’ principle), since density of jute fibre is low (1.46 g/cm^3^) and the laminate has a higher void content.

### 3.2. Mechanical Properties

The polyester showed brittle behaviour with no ability to absorb energy after cracking. Table 3 shows the results of tensile strength, tensile modulus and Poisson’s ratio for 5 specimens tested. The strength (48.64 ± 4.57 MPa), modulus (3.24 ± 0.29 GPa), and Poisson’s ratio (0.39 ± 0.04) were close to the values provided by the manufacturer.

**Figure 6:**
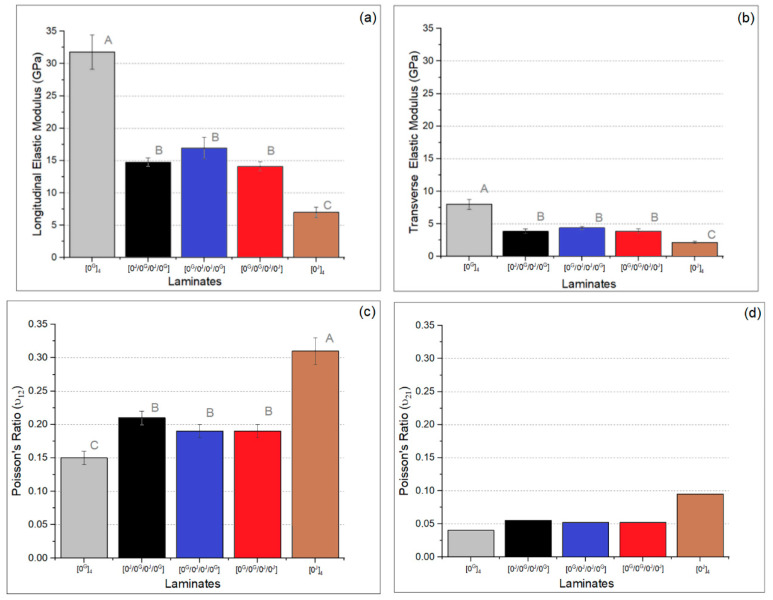
Longitudinal (**a**) and transverse (**b**) tensile modulus, major Poisson’s ratio (*ν*_12_) (**c**), and calculated minor Poisson’s ratio (*ν*_21_) (**d**) of the laminates (letters represent the results of the Tukey’s test).

Figure 6a,b shows the longitudinal and transverse tensile modulus for the five laminates. The tensile modulus and strength presented similar behaviour, with the hybrids showing intermediate properties to pure glass and pure jute laminates, and there was no significant difference among the three hybrid conditions. Figure 6c shows the major Poisson’s ratio (*ν*_12_), where the pure jute laminate presents the highest value (closer to the polyester resin), followed by the hybrids and the pure glass laminate. Again, there is no significant difference among the hybrids. The minor Poisson’s ratio (*ν*_21_) was not experimentally measured since this is generally considered not as accurate as the value obtained from the reciprocal relation based on the measured mean values of *E*_1_, *E*_2_ and *ν*_12_ for each type of laminate. The calculated minor Poisson’s ratio (*ν*_21_) is shown in Figure 6d which followed the behaviour of the major Poisson’s ratio, i.e., the hybrids presented intermediate values and pure jute presented the highest value.

**Figure 7:**
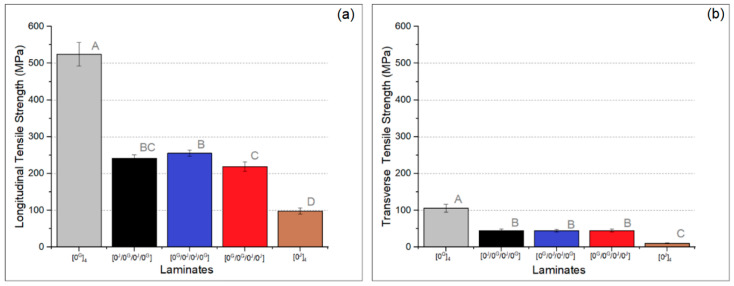
Longitudinal (**a**) and transverse (**b**) tensile strength of the laminates (letters represent the results of the Tukey’s test).

Figure 7a,b shows the longitudinal and transverse tensile strength for the laminates. The tensile strength of the pure glass laminate is nearly 5 times higher than that of the jute laminate, and the hybrids presented an intermediate behaviour. The [0^G^/0^J^/0^J^/0^G^] was slightly stronger, and this sequence is also interesting because the jute fibres are the inner layers, thus better protected against moisture absorption. The lower value for [0^G^/0^G^/0^J^/0^J^] is likely due to manufacturing problems, since this composite had the highest void content among the hybrids (Table 1), or due to the presence of a weak block (two adjacent jute layers) near the surface, where the failure is more likely to initiate.

The fibres in the transverse direction act as stress concentrators, often leading to a lower strength than the matrix itself. In the present work, only the pure jute laminate showed lower strength than the polyester resin (48.64 MPa). In Figure 1, it is clear that the jute fabric is purely unidirectional, with an expected poor transverse behaviour, whereas the used glass fabric has a small amount of fibres in the transverse direction that contribute to a small increase in strength in that direction. In the case of hybrids, the combination of reinforcements leads to a transverse strength close to that of the polyester.

Laranjeira and co-workers [39] investigated tensile properties for unsaturated polyester/jute composite manufactured by compression mould, unidirectional fibre composites with 0–50% weight fibre content and a randomly distributed short-fibre composite with 30%. The unidirectional composites were tested along and transversally to the fibre axis. Higher values for all mechanical properties were obtained when long fibre-oriented composites were tested along the fibre axis. The tensile properties in the transverse direction were dominated by the strain the fibre/matrix interface. For the 40% weight fibre content in the longitudinal direction, the tensile modulus was 5.61 ± 0.33 GPa and the tensile strength was 141.97 ± 8.76 MPa, so for the transverse direction, the tensile modulus was 1.86 ± 0.33 GPa and the tensile strength was 3.82 ± 1.33 MPa. These results were close to the present work, except for the longitudinal tensile strength, which was approximately 30% higher than the present work.

Oliveira and co-workers [40] compare the behaviour of isophthalic polyester matrix composites reinforced with unidirectional curaua fibres with that of unidirectional glass fibre composites manufactured by compression mould. The composites were produced varying in the reinforcement angle (0°, 15°, 30°, 45°, 60°, 75°, and 90°) and the fibre volumetric fraction (10%, 20%, 30%, 40%, and 50%). They found that the increase in reinforcement angle significantly decreased strength and modulus of the composites, and the glass fibre composites showed a more pronounced dependence on fibre orientation. For the 0° fibre orientation, the tensile strength of the glass laminate was 288 MPa and the tensile modulus was 20 GPa; finally, for the 90° fibre orientation, the tensile strength was 5 MPa and the tensile modulus was 4 GPa. These results were smaller than the present work, and less difference was observed for the modulus than the strength.

**Figure 8:**
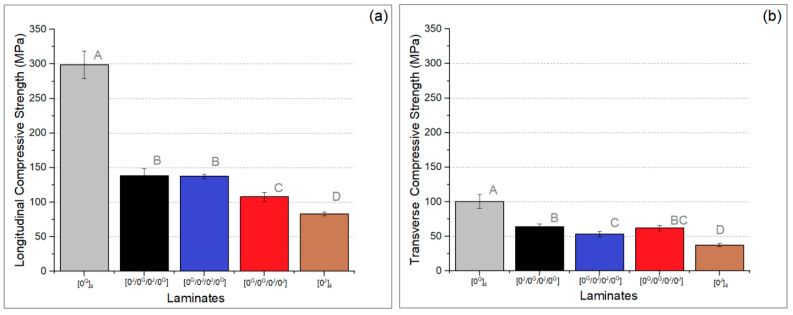
Longitudinal (**a**) and transverse (**b**) compressive strength of the laminates (letters represent the results of the Tukey’s test).

Figure 8a,b shows the longitudinal and transverse compressive strength of the laminates. The longitudinal strength is higher than the transverse strength for all laminates, as expected. Among the hybrids, the [0^G^/0^G^/0^J^/0^J^] presents the lowest longitudinal strength, as noticed before for tensile strength. For transverse strength, the hybrids showed similar behaviour.

In general, the tensile and the compressive strength tended to be higher for the [0^G^/0^J^/0^J^/0^G^] and lower for the [0^G^/0^G^/0^J^/0^J^] laminates, although the differences were not significant in most cases. This may also be partly related to the symmetry of the [0^G^/0^J^/0^J^/0^G^] laminate. In addition, the [0^G^/0^J^/0^J^/0^G^] showed the greatest homogeneity in the C-scan analysis among the hybrids, which also favours strength due to decreased likelihood of the presence of weaker regions. On the other hand, the [0^G^/0^G^/0^J^/0^J^] showed the highest contrast in the C-scan map, which may indicate greater manufacturing issues that lead to a higher void content. In addition, it is possible to observe in the micrographs that, when there were two adjacent jute layers, the bundles were better compacted, slightly reducing the overall thickness compared to the laminate with alternate jute layers ([0^J^/0^G^/0^J^/0^G^]).

**Figure 9:**
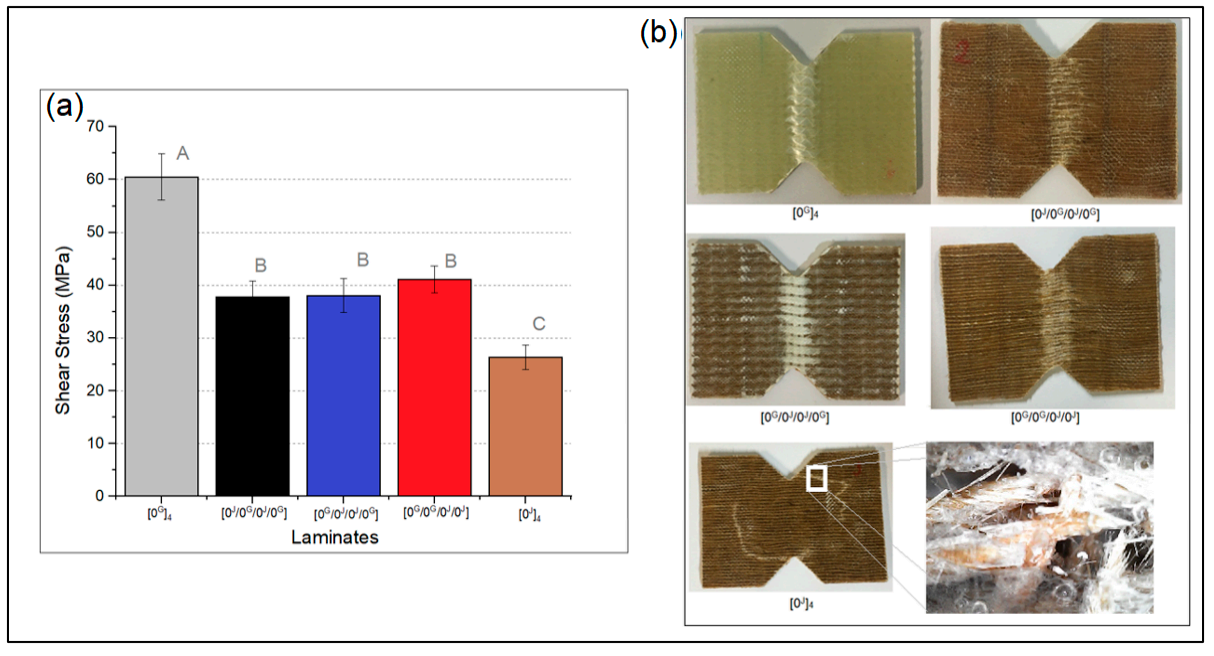
(**a**) Mean shear strength of the laminates, and (**b**) images of the specimens after testing (letters represent the results of the Tukey’s test).

Figure 9a shows the shear stress of the laminates at 5% of strain, which followed a similar behaviour to that of tensile and compressive strength, that is, the hybrids showed intermediate properties to pure glass (60.5 MPa) and pure jute (26.3 MPa) laminates, with no significant differences among the three hybrids.

As can be seen in Figure 9b, the pure glass and the hybrid laminates displayed acceptable failure morphology in the in-plane shear test, but not the pure jute laminate. The pure jute specimen must be failing in another way before shearing. In the zoomed-in image in Figure 9b, it is possible to see the failure of the matrix and the fibres holding the material, which did not happen with the other laminates.

Fajrin and co-workers [41] experimentally studied jute fibre–epoxy composites prepared by vacuum bagging process using Iosipescu shear testing (also in-plane shear); the jute fibre were provided in woven configurations. They reported a shear strength of 25.5 MPa and diagonal fractures known as off-axis failure mode. These findings were similar to those in the present work regarding the magnitude of the shear strength and the presence of diagonal fractures.

The engineering constants of the [0^G^/0^J^/0^J^/0^G^] could be estimated using laminate plate theory [34], since this hybrid laminate is symmetrical and therefore its [B] matrix is null. Table 3 compares the experimental and analytical values for this laminate, and a very good agreement is noticed, indicating good overall consistency in the results.

Torabizadeh [42], experimentally investigated the tensile, compressive and in-plane shear behaviour of unidirectional glass fibre laminate manufactured by hand lay-up method, used epoxy resin as matrix and the found the fibre volume fraction to be 55%. The tensile test was performed according to ASTM D3039, the compression test according to ASTM D3410, the in-plane shear test followed the ASTM A4255 (three-rail shear test method). In tensile, the laminate exhibits a linear elastic behaviour until breakage; for the longitudinal, the tensile modulus was 22.67 GPa, and the tensile strength was 715.13 MPa; for the transverse, the tensile modulus was 5.58 GPa, and the tensile strength was 69.04 MPa. For the compression test, the stress–strain relation was slightly nonlinear and final failure occurred catastrophically in all cases. The compressive strength in longitudinal direction at room temperature was 570.37 MPa, and in the transverse direction was 122.12 MPa. For the in-plane shear, the stress–strain behaviour was highly nonlinear; this nonlinear behaviour was attributed to the micro-crack accumulation throughout the matrix. The in-plane shear strength found was 68.11 MPa, and the shear modulus was 2.14 GPa.

It is possible to observe in the work of Torabizadeh [42] that the tensile strength was higher than compressive strength at the longitudinal; the opposite occurs at the transverse direction. The same happened in the present work, but in the transverse the tensile strength and the compressive strength were very close for pure glass laminate. For the longitudinal, the tensile strength in the present work was smaller, and in the transverse it was a little higher. The tensile modulus in the present work was slightly higher. The transverse compressive strength presented a good agreement. Finally, the tensile stress found in both works were close, regardless of different test methods used.

Zhang and co-workers [43] investigated the effect of the stacking sequence on the mechanical properties of unidirectional flax/glass fibre-reinforced hybrid composites. It was observed that the tensile modulus was almost the same for all hybrids. However, the stacking sequence showed a greater influence on tensile strength and failure strain. The hybrid with alternated layers showed the highest values among the hybrids, followed by the hybrid with glass at the ends and that with adjacent fibres.

A similar behaviour found in Zhang and co-workers [43] was observed in the present work, i.e., no significant differences in tensile modulus for the hybrids. However, strength was more affected, and the hybrids with more layer interaction between jute and glass fibre plies ([0^J^/0^G^/0^J^/0^G^] and [0^G^/0^J^/0^J^/0^G^]) led to higher tensile strength, and also compressive strength. Therefore, in addition to manufacturing issues, this may partly justify the lower properties of the [0^G^/0^G^/0^J^/0^J^].

Abdullah-Al-Kafi and co-workers [44] investigated jute fibre (Hessian cloth) and E-glass fibre (mat)-reinforced with unsaturated polyester resin prepared by the hand lay-up technique. For the pure jute laminate, they found that as the fibre content (%wt) increases, the stress is more evenly distributed, and the strength of the composites increases up to 25% in jute content; after that, the composite exhibits a decreased value over the fibre content. For the hybrids, the addition of glass fibre to a jute-based composite usually showed a more positive hybrid effect by the significant improvement in mechanical properties than that of the unhybridized. In a hybrid composite, the mechanical properties of composites are mainly dependent on the modulus and percentage elongation at break of individual reinforcing fibres. The increase in mechanical properties through the addition of glass fibre to jute can be explained on the basis of higher modulus and elongation at break of the glass fibre [45], whereas the extensibility of glass is low compared to the jute fibre. This results in early glass fibre failure, which transfers high stress to the jute fibres, and also in the failure of composites [46]. In the present work, the hybridization also had a positive effect in the mechanical properties.

## 4. Conclusions

This study investigated the effect of hybridization and stacking sequence on the mechanical and physical characteristics of five laminates, namely pure jute, pure glass and three hybrid configurations. Based on the results, the following conclusions can be drawn:

The [0^J^/0^G^/0^J^/0^G^] and [0^G^/0^G^/0^J^/0^J^] laminates showed greater colour contrast and higher concentration of bright spots in the C-scan analysis, indicating lower overall quality and homogeneity than the [0^G^/0^J^/0^J^/0^G^] laminate. The pure glass laminate presented the highest fibre content (54.1%), and pure jute presented the highest matrix (56.4%) and void (15.5%) content. The contents of void and fibre were similar among the hybrids, being slightly higher for the [0^G^/0^G^/0^J^/0^J^], corroborating the C-scan analysis. The glass laminate presented higher density, followed by the hybrids and the jute laminate, as expected considering the higher density of the glass fibres.

The longitudinal properties were higher than transverse properties for all laminates, as expected. The hybrids did not present significant difference in tensile modulus. The longitudinal tensile strength of the [0^G^/0^J^/0^J^/0^G^] was slightly higher than the other hybrids, which was related to the symmetry of this laminate and more fibre layer interaction between jute fibre plies and glass fibre plies. In addition, this laminate showed the greatest homogeneity in resin distribution among the hybrids, which also favours strength due to decreased likelihood of the presence of weaker regions. Among the hybrids, the [0^G^/0^G^/0^J^/0^J^] presented the lowest longitudinal tensile strength probably due to manufacturing issues or the presence of two adjacent jute layers near the surface that may behave as weaker region.

The compressive strength followed a similar behaviour of tensile strength. Therefore, in addition to possible manufacturing issues, the presence of a block of two jute layers at the surface of the laminate may have led to premature failure and lower properties for the [0^G^/0^G^/0^J^/0^J^]. Regarding shear stress at 5% of strain, the hybrids showed intermediate values to pure glass (60.5 MPa) and pure jute (26.3 MPa) laminates, with no significant differences among the three hybrids.

In all, the present work showed that hybridization of jute with glass fibres led to improved properties compared to the pure jute composite, and the stacking sequence did not lead to great differences in the evaluated properties of the hybrids. The studied hybrid laminates showed potential in substituting glass fibre composites in moderate applications, contributing to the environment by reducing the amount of synthetic fibres in composites.

## Figures and Tables

**Table 1:** Volume fraction of the constituents in the laminates.

	[0^G^]_4_	[0^J^/0^G^/0^J^/0^G^]	[0^G^/0^J^/0^J^/0^G^]	[0^G^/0^G^/0^J^/0^J^]	[0^J^]_4_
*V_J_* (%)	0.00	17.00	17.71	17.51	28.00
*V_G_* (%)	54.14	20.00	20.69	20.46	0.00
*V_f_* (%)	54.14	37.00	38.40	37.97	28.00
*V_m_* (%)	37.31	51.98	49.84	49.03	56.41
*V_v_* (%)	8.55	11.02	11.76	13.00	15.59

**Table 2:** Thickness of each layer of the laminates, in mm.

Layers	[0^G^]_4_	[0^J^/0^G^/0^J^/0^G^]	[0^G^/0^J^/0^J^/0^G^]	[0^G^/0^G^/0^J^/0^J^]	[0^J^]_4_
1	0.31	0.61	0.27	0.30	0.66
2	0.38	0.32	0.54	0.22	0.56
3	0.33	0.56	0.65	0.60	0.47
4	0.31	0.33	0.28	0.64	0.51
Overall	1.33	1.82	1.74	1.76	2.20

**Table 3:** Experimental and analytical properties of the [0^G^/0^J^/0^J^/0^G^] laminate.

	*E_x_* (MPa)	*E_y_* (MPa)	*G_xy_* (MPa)	$v_{xy}$	$v_{yx}$
Experimental	16.93 ± 1.68	4.36 ± 0.54	---	0.19 ± 0.01	0.08 ± 0.005
Analytical	16.30	4.34	3.76	0.20	0.05

## Data Availability

Not applicable.
